# Genome-wide association study for stayability at different calvings in Nellore beef cattle

**DOI:** 10.1186/s12864-024-10020-y

**Published:** 2024-01-23

**Authors:** Diogo Osmar Silva, Gerardo Alves Fernandes Júnior, Larissa Fernanda Simielli Fonseca, Lúcio Flávio Macedo Mota, Tiago Bresolin, Roberto Carvalheiro, Lucia Galvão de Albuquerque

**Affiliations:** 1 Animal Science Department, School of Agricultural and Veterinary Sciences, São Paulo State University (Unesp), Jaboticabal, SP Brazil; 2 National Council for Scientific and Technological Development (CNPq), Brasília, Brazil; 3 Present address: Departamento de Zootecnia, Via de acesso Paulo Donato Castellane s/n., São Paulo, Jaboticabal CEP: 14884-900 Brazil

**Keywords:** Reproductive trait, Single-step, Zebu breed

## Abstract

**Backgrounding:**

Stayability, which may be defined as the probability of a cow remaining in the herd until a reference age or at a specific number of calvings, is usually measured late in the animal’s life. Thus, if used as selection criteria, it will increase the generation interval and consequently might decrease the annual genetic gain. Measuring stayability at an earlier age could be a reasonable strategy to avoid this problem. In this sense, a better understanding of the genetic architecture of this trait at different ages and/or at different calvings is important. This study was conducted to identify possible regions with major effects on stayability measured considering different numbers of calvings in Nellore cattle as well as pathways that can be involved in its expression throughout the female’s productive life.

**Results:**

The top 10 most important SNP windows explained, on average, 17.60% of the genetic additive variance for stayability, varying between 13.70% (at the eighth calving) and 21% (at the fifth calving). These SNP windows were located on 17 chromosomes (1, 2, 4, 6, 7, 8, 9, 10, 11, 12, 13, 14, 18, 19, 20, 27, and 28), and they harbored a total of 176 annotated genes. The functional analyses of these genes, in general, indicate that the expression of stayability from the second to the sixth calving is mainly affected by genetic factors related to reproductive performance, and nervous and immune systems. At the seventh and eighth calvings, genes and pathways related to animal health, such as density bone and cancer, might be more relevant.

**Conclusion:**

Our results indicate that part of the target genomic regions in selecting for stayability at earlier ages (from the 2th to the 6th calving) would be different than selecting for this trait at later ages (7th and 8th calvings). While the expression of stayability at earlier ages appeared to be more influenced by genetic factors linked to reproductive performance together with an overall health/immunity, at later ages genetic factors related to an overall animal health gain relevance. These results support that selecting for stayability at earlier ages (perhaps at the second calving) could be applied, having practical implications in breeding programs since it could drastically reduce the generation interval, accelerating the genetic progress.

## Background

Reproductive efficiency in beef cattle is one of the main factors defining the number of animals available for selection and slaughter. It helps to explain why reproductive traits have, generally, the greatest importance in economic selection indexes. Brumatti et al. [1], for example, verified that reproductive traits have between four and thirteen times more economic impact than growth traits and, stayability being one of the most important traits on beef herd profitability. Stayability (STAY) may be defined as the probability of a cow remaining in the herd until a reference age or a specific number of calving, since it had the opportunity to reach that age or calving number [2]. Indeed, the maintenance of cows and heifers for replacement is responsible for a large part of the cost in beef cattle production systems [3] and this cost increases with low reproductive rates [4]. Increasing female’s productive longevity can reduce heifer replacement costs [5]. The idea is that the cow must remain in the herd at least to pay its raising and maintenance cost, and STAY is a common trait used to measure the period that the female remains productive in the herd.

Selection for STAY is difficult because it usually presents low heritability, ranging between 0.08 and 0.14 [6, 7], in addition to be a sex-limited trait measured late in female’s life, normally at 76 months of age. All these factors decrease the expected genetic gain when this trait is included as selection criterion. Therefore, selecting for STAY at earlier ages would be an alternative to increase the rate of the genetic gain for this trait, with the hypothesis that most of the genetic factors with major effects on this trait act throughout female’s productive life.

It is worth mentioning that some genome-wide association studies have already been applied to identify genetic variants associated to STAY in cattle [6, 8, 9], but, to our knowledge, none considered STAY at different calvings. Therefore, this study was conducted to identify possible genomic regions and pathways related to genes with effects on the expression of STAY throughout female’s productive life, in a commercial Nellore population. The results of this study may provide a better understanding of the expression of this trait at different stages of animal life.

## Materials and methods

### Phenotypic dataset

Information of 2,084,880 Nellore animals, of which 672,000 were dams, born between 1984 and 2017 and belonging to 583 farms and three breeding programs (DeltaGen, Paint and CIA de Melhoramento) were utilized in the present study. In the reproductive management, females between fourteen and eighteen months of age participate in their first breeding season for 60 days in order to identify sexually precocious females. The heifers that did not conceive, non-precocious, have another chance to breeding when they are 22 months of age, on average. But if failing to calve after this second breeding season, these non-precocious heifers are culled.

STAY was evaluated from the second to the eighth calving. Only females with first calving until 40 months of age were phenotyped for STAY. For each calving, STAY was a binary trait in which the value 2 was attributed for females that had the respective calving and 1 for those that had the opportunity of mating but did not calve. If the interval between the date of the last calving of a female and the date of the last calving recorded in the dataset was smaller than 500 days, the subsequent records for that female were considered as missing. This is important to take into account that young females did not have the opportunity to express their phenotypes for STAY at older ages. More details with examples about phenotypic definition are available at Morales et al. [10].

Contemporary groups (CG) were defined as: herd, year and season of birth, management group at weaning and at yearling and precocity score. The birth seasons were defined as dry (March to August) and rainy (September to February) and the precocity scores were: 2 (precocious) for cows with age at first calving until 31 months and 1 (non-precocious) for females with age at first calving higher than 31 months. CGs without variability as well as with less than three animals were excluded. The percentage of precocious females in each measure of STAY was 34.4%, 23.3%, 18.4%, 17.0%, 15.6%, 14.5% and 15.5% for the second, third, fourth, fifth, sixth, seventh and eighth calving, respectively.

The final database used in the analyses had 195,452; 161,261; 130,236; 103,043; 79,844; 62,663 and 47,045 females with phenotypic information for the second; third; fourth; fifth; sixth; seventh and eighth calving, respectively. The percentage of females with precocity score of 2 (success) for STAY was 50.7%, 33.0%, 23.2%, 17.2%, 13.4%, 10.5% and 8.6%, for second to eighth calving, respectively.

### Genotypic dataset

In total, 2,021 females and 949 sires had genotyped information from a panel with 777,962 SNP markers (Illumina BovineHD BeadChip). All SNPs were mapped according to the ARS-UCD1.2 reference map [11]. Females with genotypic information were born between 2007 and 2013 and the sires between 1993 and 2011. The genotype quality control was performed considering only autosomal markers and following SNP marker exclusion criteria: MAF lower than 2%; call rate lower than 0.90; highly correlated SNPs (*r* ^2^ > 0.995) and markers that show deviation of heterozygosity higher than 0.15 in comparison to the expected heterozygosity according to the Hardy-Weinberg equilibrium. In addition, animals with call rate lower than 0.90 were excluded. After quality control, 3.849 genotyped animals and 474.640 markers remained. All data editing was performed using R software version 4.3 [12].

### Genome-wide association studies

Stay at different calving’s were evaluated through a single-trait generalized linear mixed animal model considering a probit function using the program THRGIBBS1F90 from blupf90 suite [13]. The general model can be described as follows:

$$\boldsymbol n=\mathbf\chi\boldsymbol\beta+\mathbf W\boldsymbol\alpha+\boldsymbol e,$$

where **ɳ** represents the linear predictor; ***β*** is a vector of fixed effect of CG; ***a*** is a vector of animal additive effect, assuming *a* ~ N(0, **H** ${\sigma }_{a}^{2}$), where **H** is a relationship matrix that combines pedigree (**A**) and genomic information (**G**) and ${\sigma }_{a}^{2}$ corresponds to the additive genetic variance; ***e*** is a vector of residuals, assuming *e* ~ N (0, **I** ${\sigma }_{e}^{2}$), where **I** is an identity matrix and ${\sigma }_{e}^{2}$ is the residual variance. The $\mathbf{X}$, and **W** are incidence matrices relating $\mathbf{y}$ to the fixed effects (***β***), and to the additive genetic effects ($\mathbf{a}$), respectively.

The inverse of the **H** matrix used to solve the mixed model equations was obtained according to [14]:

$$\mathbf{H}^{-1} = \mathbf{A}^{-1}\left[\begin{array}{cc}0& 0\\ 0& {{\mathbf{G}}^{-1}-\mathbf{A}}_{22}^{-1}\end{array}\right],$$

where ${\mathbf{G}}^{-1}$ corresponds to the inverse of the genomic relationship matrix and ${\mathbf{A}}_{22}^{-1}$ is the inverse of the pedigree relationship matrix for the genotyped animals. The G matrix was obtained according to [15]:

$$\mathbf G\boldsymbol=\mathbf{ZDZ}'q,$$

where **Z** is a matrix of genotypes adjusted for allele frequencies; **D** is a diagonal matrix with weights for SNP effects assuming; and *q* is a normalization factor.

Variance components were estimated by Bayesian inference via Gibbs sampling. A total of 300,000 Gibbs samples were generated, considering a burn-in of 30,000 iterations and samples being stored every ten iterations. The convergence analysis was verified through graphic inspection as well as based on the [16] and [17] tests, using the boa package version 1.1.8 [18].

The SNP effects were obtained using the postGSf90 software [19], following the method described in [20]:

$${\hat{\mathbf{u}}_{\mathbf{s}}=\mathbf{D}{\mathbf{Z}}^{\mathbf{{\prime }}}\left[\mathbf{Z}\mathbf{D}\mathbf{Z}\varvec{{\prime }}\right]}^{-1}\hat{\mathbf{a}}$$

where, $\hat{\mathbf{u}}_{\mathbf{s}}$ is a vector with SNP effects, **D** is a diagonal matrix containing the weights for each SNP marker; **Z** is a matrix with genotypes; $\hat{\text{a}}$ is the vector of genomic estimated breeding values (GEBV) for genotyped animals. The SNP effects estimation was performed considering two iterations. In the first iteration, **D** was an identity matrix. In the second iteration, the **D** matrix was updated with weights (${d}_{i}$) calculated in the first iteration as in [21]: ${d}_{i} = \hat{\text{u}}_{i}^{2}*2{p}_{i} \left(1- {p}_{i}\right)$, where $\hat{\text{u}}_{i}^{2}$ is the square of the effect for the i^th^ SNP and ${p}_{i}$ is the frequency of the second allele of the i^th^ SNP. The GEBV (â) were also updated for the second iteration [20].

Genomic regions with major effects on STAY at different calving were identified based on the proportion of variance explained by windows with 100 consecutive SNPs. The proportion of genetic variance explained by each SNP-window was calculated as follow:

$$\frac{Var \left({a}_{i}\right)}{{{\sigma }_{a}}^{2}} x 100\%= \frac{Var\left({\sum }_{j=1}^{100}{Z}_{j}\hat{\text{u}}_{j}\right)}{{\sigma }_{a}^{2}} x 100 \%$$

where ${a}_{i}$ is the genetic value of the i^th^ SNP-window; ${{\sigma }_{a}}^{2}$ is the total genetic variance; ${Z}_{j}$ is the vector with genotype for the j^th^ SNP for all animals; and $\hat{\text{u}}_{j}$ is the estimated effect for each marker within the SNP-window.

The identification of genes in the top ten SNP-windows with the highest percentage of the additive genetic variance was performed using the program ENSEMBL [22], considering the *Bos Taurus* ARS-UCD1.2 assembly [11] as reference. The list of genes was then submitted to the software DAVID v6.7 [23] to identify over-represented gene ontology terms and pathways associated with STAY. This enrichment analysis was done considering a *p*-value < 0.05 as significant.

## Results and discussion

The heritability estimates were 0.17, 0.15, 0.17, 0.17, 0.16, 0.17 and 0.17 for second, third, fourth, fifth, sixth, seventh and, eighth calving, respectively. These values are similar or lower to those reported in the literature for stayability measured at 76 months in Nellore (0.14) and Nellore-Angus crossbred (0.12) [24, 25]. Our estimates are higher than those obtained for stayability in consecutive calvings in Hereford cattle (from 0.05 to 0.08), without genomic information [26].

**Fig. 1:**
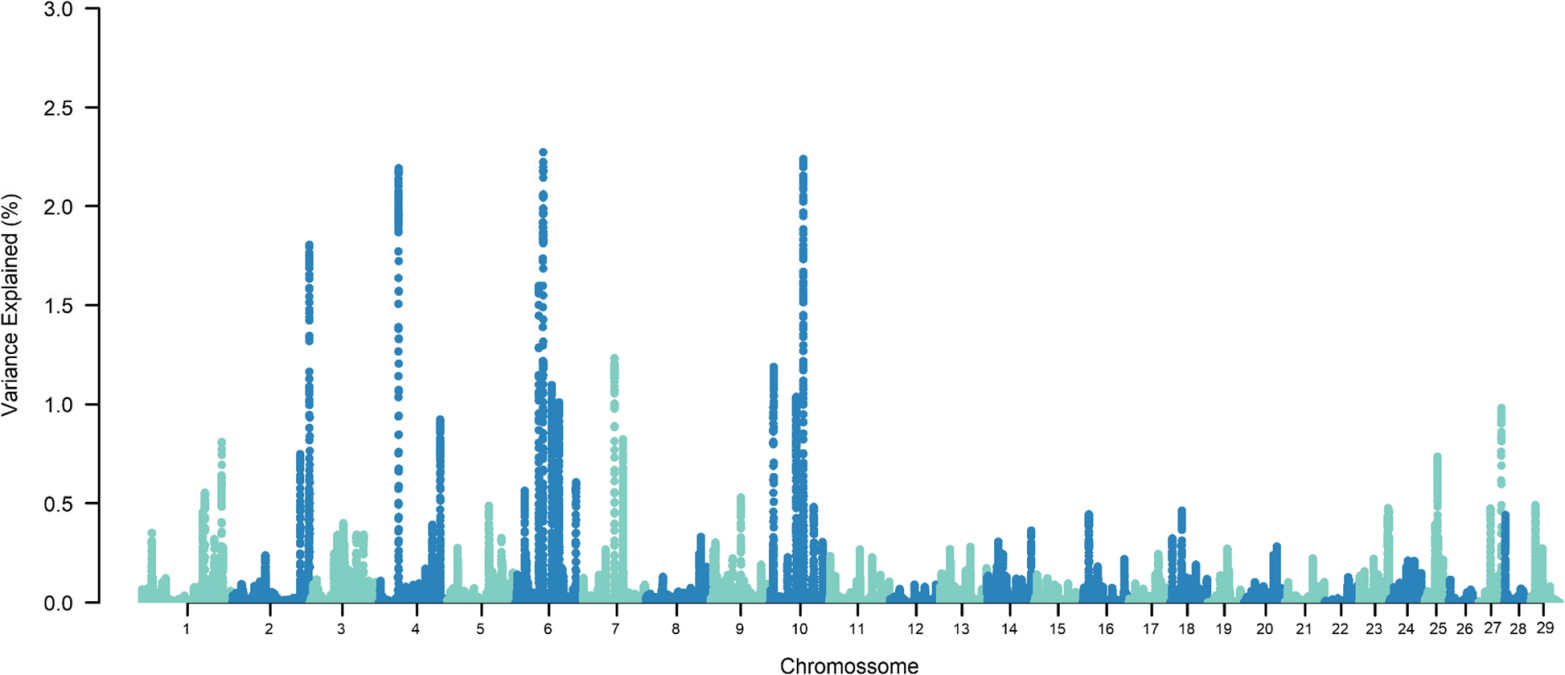
Manhattan plot for stayability measured at second calving in Nelore cattle

**Fig. 2:**
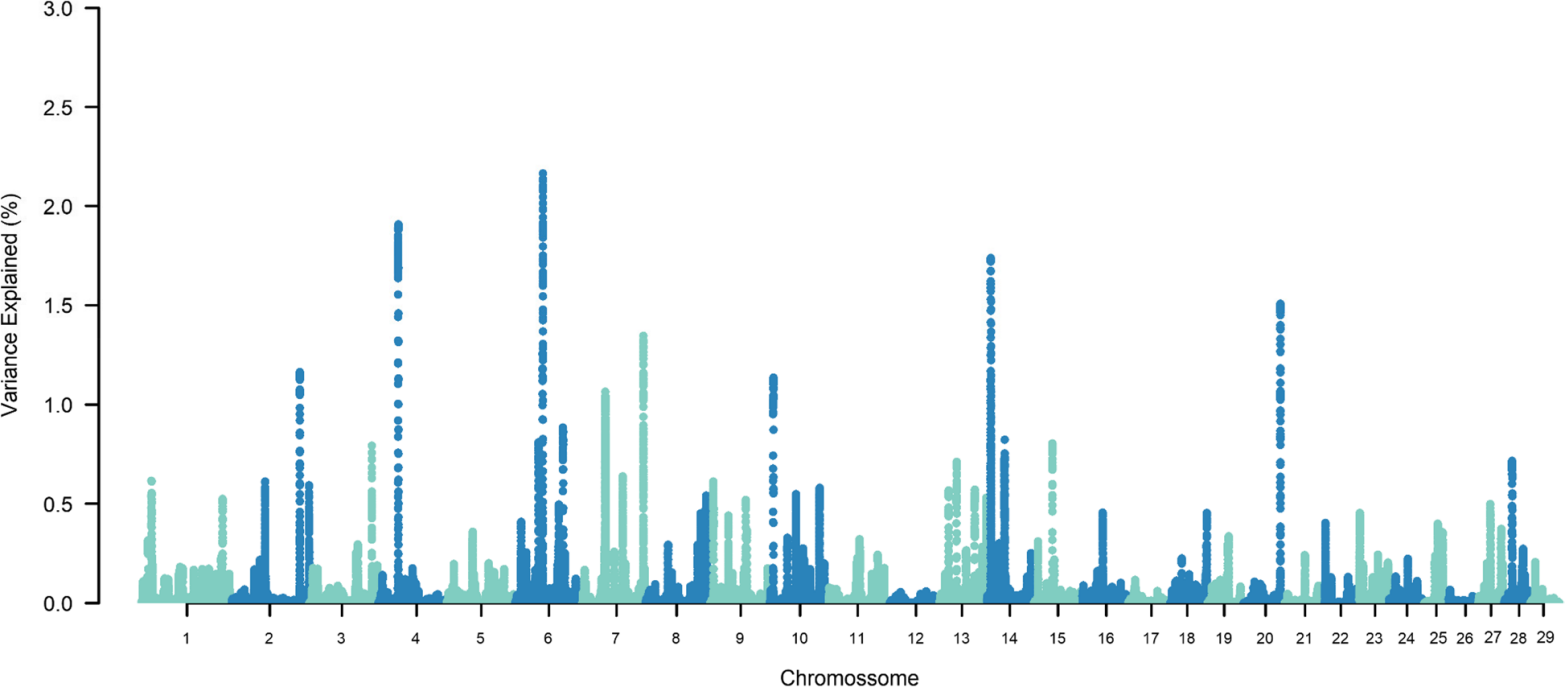
Manhattan plot for stayability measured at third calving in Nelore cattle

**Fig. 3:**
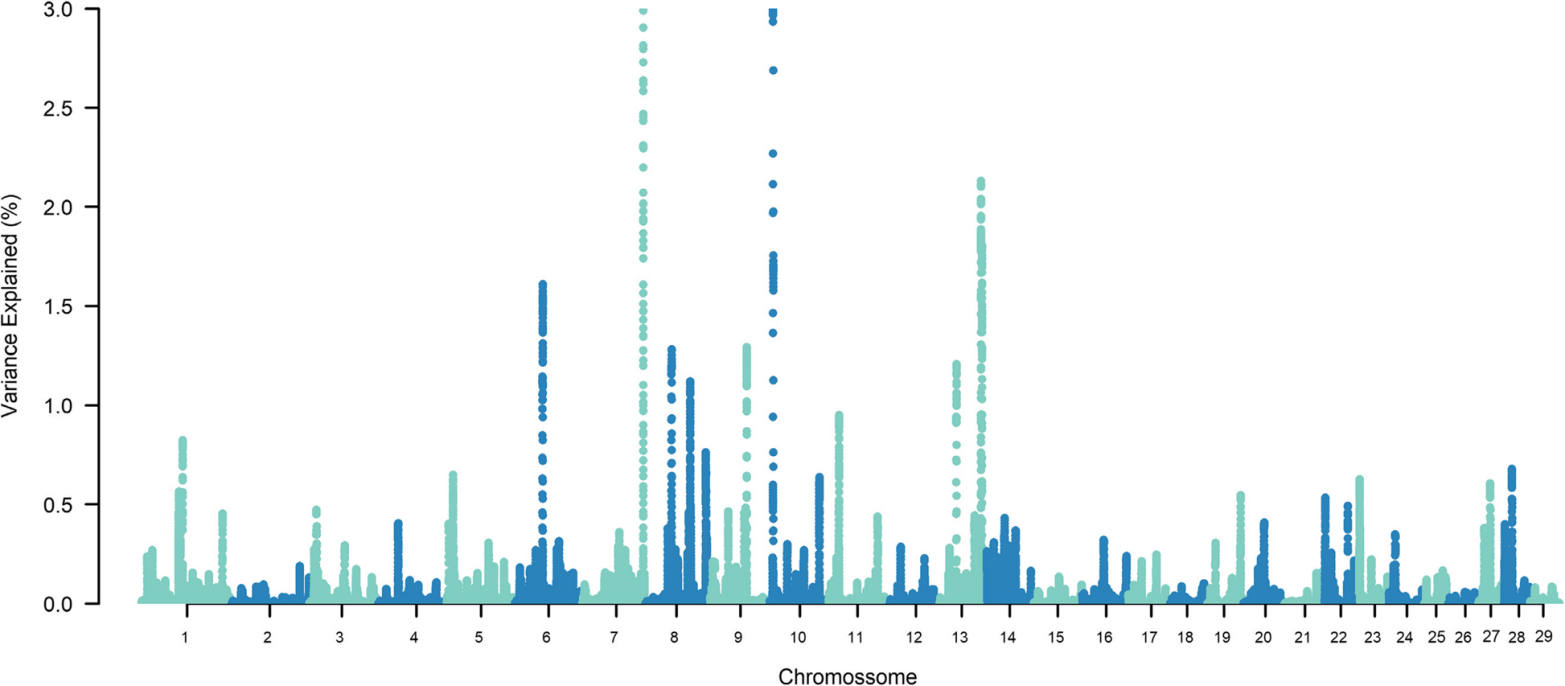
Manhattan plot for stayability measured at fourth calving in Nelore cattle

**Fig. 4:**
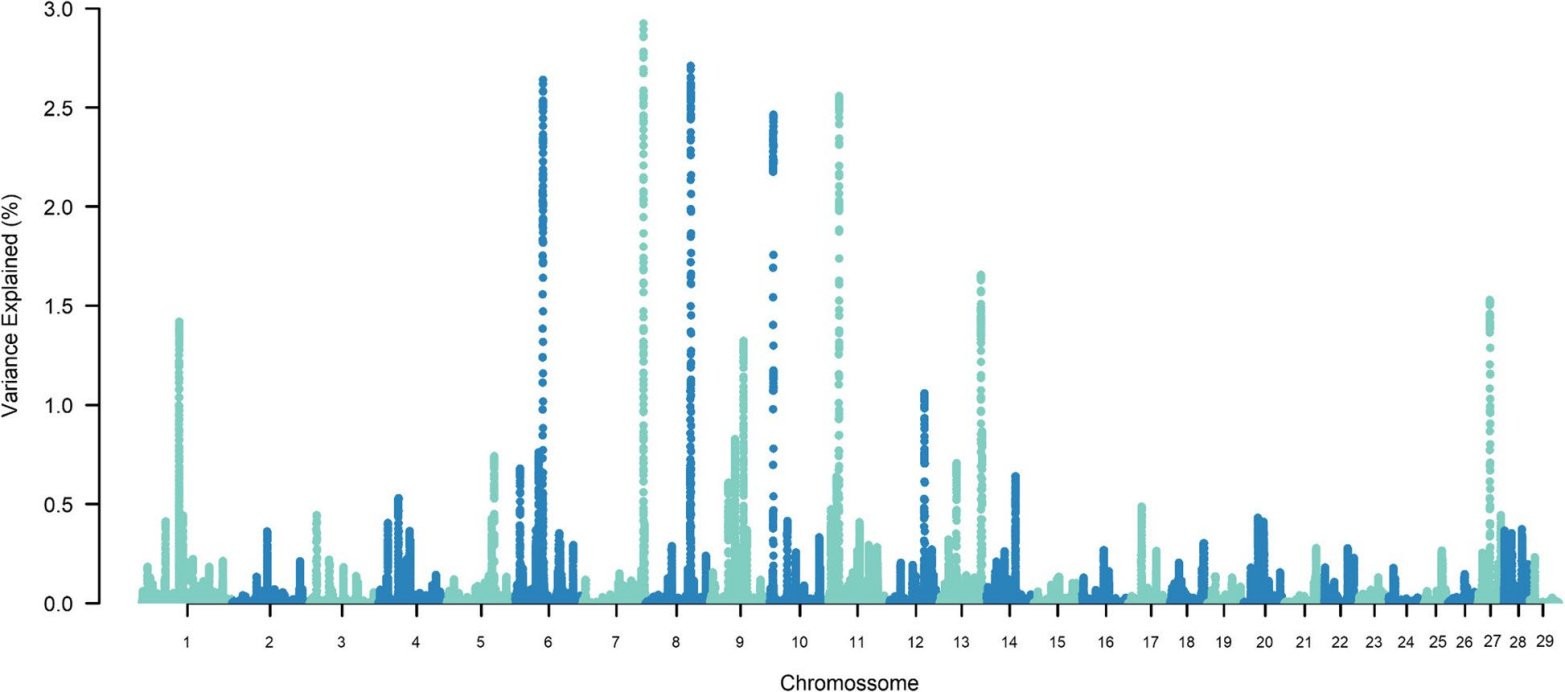
Manhattan plot for stayability measured at fifth calving in Nelore cattle

**Fig. 5:**
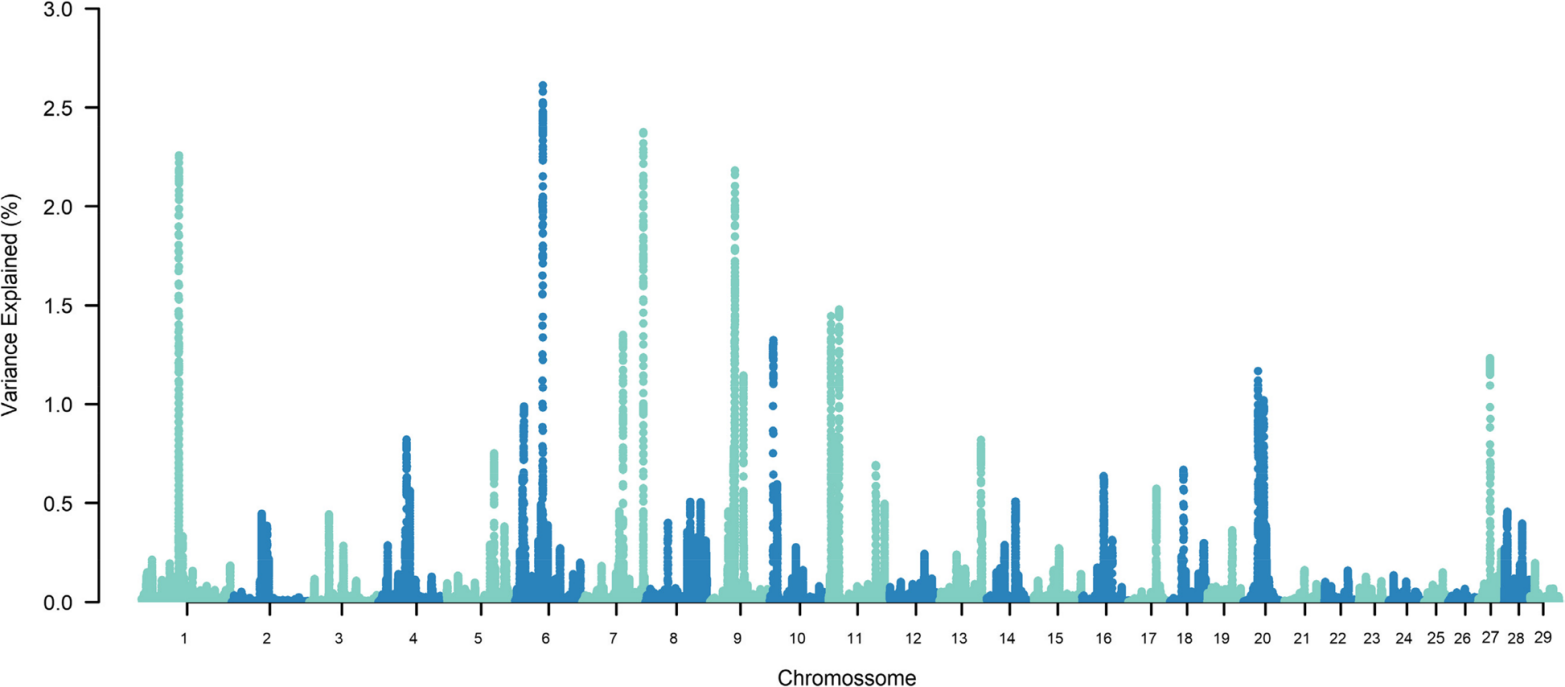
Manhattan plot for stayability measured at sixth calving in Nelore cattle

**Fig. 6:**
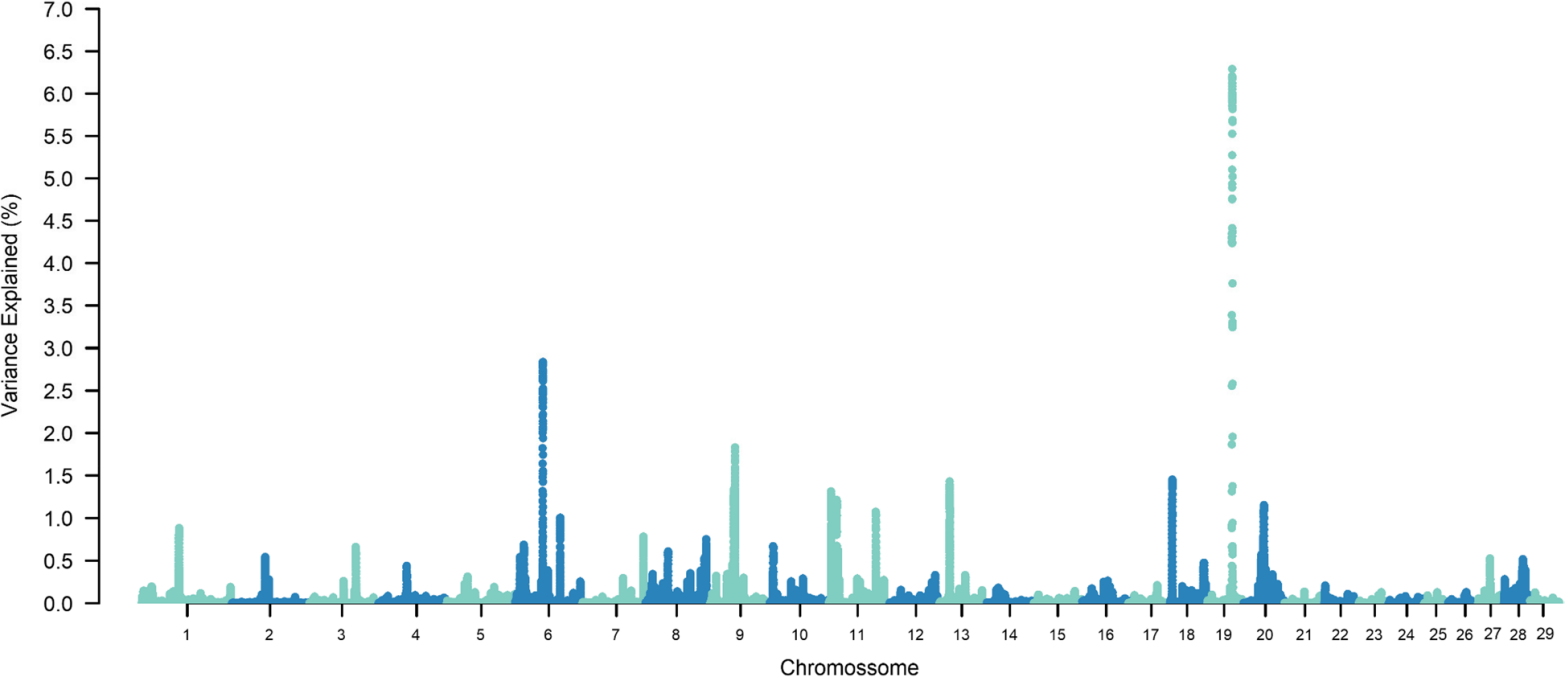
Manhattan plot for stayability measured at seventh calving in Nelore cattle

**Fig. 7:**
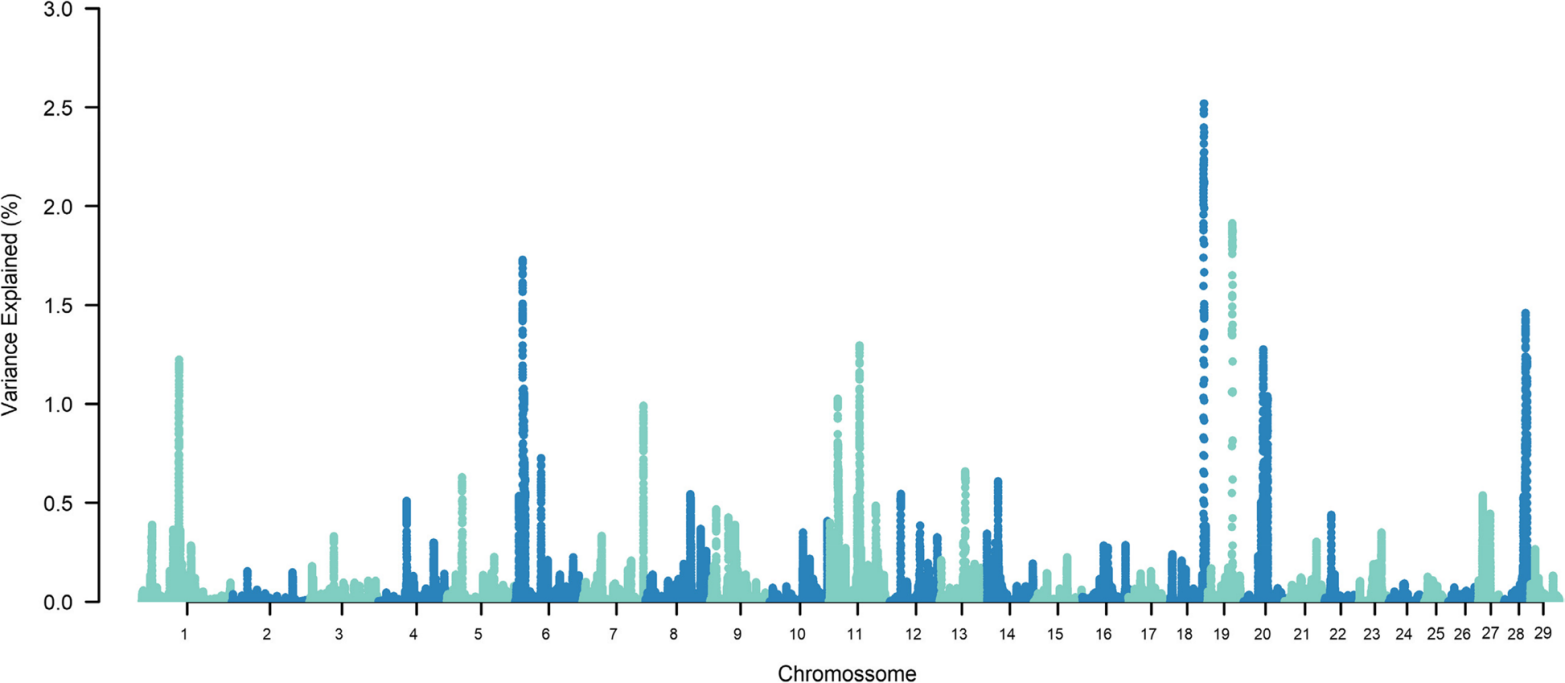
Manhattan plot for stayability measured at eighth calving in Nelore cattle

The top ten SNP-windows explained, on average, 17.60% of the genetic additive variance for STAY, varying between 13.70% and 21% according to the calving considered (Figs. 1, 2, 3, 4, 5, 6 and 7). The major SNP-windows are located on chromosomes 1, 2, 4, 6, 7, 8, 9, 10, 11, 12, 13, 14, 18, 19, 20, 27, and 28. Some of these chromosomes have been already reported by Teixeira et al. [24] in Nellore and Speidel et al. [25] in Angus, for STAY at a specific age. Teixeira et al. [24] reported genomic regions associated with STAY at 65 months of age on chromosomes 1, 2, 5, 6, 9, 20, and X. Speidel et al. [25], defining STAY of the female to produce 5 consecutive calves by 6 years of age, reported SNPs on chromosomes 6, 8, 9, 12, 15, 18, 22, and 23.

A total of 176 genes, associated with STAY, were identified (Table 1) and some regions were reported in other studies for STAY at a specific age. For instance, the BTA9:40.7–41.7 Mb region, identified here for the seventh calving, overlaps with Teixeira’s [23]. Also, the BTA6:13.0-13.4 Mb, BTA8:45.9–46.2, and BTA18:59.3–60.4 Mb regions identified for the eighth, fourth and eighth calvings, respectively, are very close (distance < 1 Mb) to SNP regions reported by Speidel et al. [25]. It is worth mentioning that caution should be taken in comparisons between our and the aforementioned studies due to differences on STAY definitions, methodology of analysis and also genome reference. In both studies, [24] and [25], the reference genome was the UMD 3.1, an earlier and less accurate assembly than the ARS-UCD1.2 used in the present study [27].

**Table 1:** Chromosome (BTA), region and candidate genes with known function for stayability in different calvings for Nellore cattle

BTA	Region (bp)	Gene	Calving
1	65778705–66049468	STXBP5L, POLQ	5,6,8
2	119059784–119392670	ARMC9, B3GNT7, NCL, SNORA75, SNORD20, NMUR1	3
2	135494699–136128479	PADI2, ATP13A2, MFAP2, MFAP2, CROCC, NECAP2, SPATA21, SZRD1, FBXO42, CPLANE2, ARHGEF19, EPHA2, bta-mir-12026-1, FAM131C, HSPB7, SRARP	2
4	34929131–35595146	SEMA3D	2,3
6	13095116–13451538	ALPK1, TIFA, AP1AR, FAM241A	8
6	15000228–15511470	SNORA70, ELOVL6	8
6	40778768–41197646	KCNIP4	2
6	48275442–48798053		2,3,4,5,6,7
6	63473477–64175368		2
6	76329318–76933125		2
6	83689130–84224086	TMPRSS11F, TMPRSS11BNL, NAP1L1, TMPRSS11E, YTHDC1	3
7	5566681–56208813		2
7	40211447–40591774	OR2V1, TRIM7, TRIM41, RACK1, SNORD96, TRIM52, IFI47, ZNF496	3
7	71098162–71429016		6
7	106812603–107087569	FBXL17	3,4,5,6
8	45925570–46251778	C8H9orf135, MAMDC2	4
8	78390507–79466530	AGTPBP1, TBX18, ISCA1, TUT7, NAA35, GOLM1	4,5
9	41317786–41863663	FOXO3, AFG1L	7
9	43384710–43679905	RTN4IP1, CRYBG1, ATG5	6,7
9	58457036–58925654	7SK	5
9	64368103–64791347		4
10	5968117–6301733		2,3,4,5,6
10	46001413–46531091	DAPK2, HERC1, FBXL22, USP3	2
10	58079955–58662249	MYO5C, GNB5, MAPK6, LEO1, TMOD3, TMOD2, LYSMD2, SCG3	2
11	4198343–4625501	TSGA10, C2orf15, LIPT1, MITD1, MRPL30, TXNDC9, EIF5B, REV1, bta-mir-2285ac	6,7
11	53942316–54543001		8
11	82486734–83003817	DDX1, NBAS, SCGB1C1, ODF3, BET1L, RIC8A, SIRT3, PSMD13, COX8B, NLRP6, PGGHG, IFITM5	7
11	14086549–14645715	FAM136A, XDH, SRD5A2, MEMO1	7
11	18254146–18759465		4,5,6
12	60701633–61426462	HTATSF1	5
13	18256450–18625154	CREM, CUL2, PARD3	7
13	30250975–30855161	MINDY3	4
13	72996969–73347371	PKIG, ADA, CCN5, KCNK15, RIMS4, YWHAB, PABPC1L, TOMM34, STK4	4,5
13	75241356–75719626	OCSTAMP, SLC13A3, TP53RK, SLC2A10, EYA2	4
14	30933520–31352050	SGK3, MCMDC2, SNORD87, TCF24, PPP1R42, COPS5, CSPP1 ARFGEF1	3
14	6901852–7286960	bta-mir-30d, bta-mir-30b, ZFAT	3
18	3997169–4337618		7
18	59330771–60424701	ZNF677	8
19	43400368–44351789	DHX8, ETV4, MEOX1, SOST, DUSP3, CFAP97D1, MPP3, CD300LG, MPP2, PPY, PYY, PYY2, NAGS, TMEM101, LSM12, G6PC3, HDAC5, HROB, ASB16, TMUB2, ATXN7L3, UBTF, SLC4A1, RUNDC3A, SLC25A39, GRN, FAM171A2, ITGA2B, GPATCH8, FZD2	7,8
20	25330894–25766537	NDUFS4, FST	6
20	34138855–34826891		8
20	35324767–36014615	FYB1, RICTOR, OSMR, LIFR, EGFLAM	7
20	41696563–42157220	PDZD1, C20H5orf22, DROSHA	8
20	63971737–64281356	SEMA5A,	3
27	21318719–21871062	TUSC3	5,6
28	37535099–37818918	NRG3	8
28	40412640–40627853	GRID1	8

Many regions were associated with STAY in more than one calving (Table 1), indicating that part of the genetic factors may influence the expression of STAY throughout female’s productive life. This corroborates the results observed in Nellore [28] and in Canadian Simmentals [29], where moderate to high genetic correlation estimates between STAY at different calvings were found.

The BTA6:48.27–48.79 Mb region was associated with STAY from second to seventh calving (Table 1). A search in the QTL database [30] showed that this region harbors QTLs affecting average daily gain, mature weight, body temperature, fertility, and resistance to ectoparasites. Additionally, this genomic region was associated with reproduction and milk yield traits in N’Dama cattle [31]. The gene *KCNIP4* (Potassium Voltage-Gated Channel Interacting Protein 4), located in the BTA6:40–42 Mb SNP-window, was associated with calving ease in Holstein cows [32]. So, our results show that the BTA6:42-49 Mb might affect STAY throughout female’s productive life indicating that this genomic region has pleiotropic effects on adaptive and reproductive traits.

The BTA11:14-19 Mb was associated with STAY in the fourth, fifth, sixth and seventh calving (Table 1). This region shows QTLs associated with calving ease and daily weight gain. This genomic region contains the *MEMO1* (mediator of cell motility 1) gene which has been associated with somatic cell count in Holstein cattle [33]. The *MEMO1* gene, mediated by the c-Src (Src) kinase, controls the Estrogen Receptor alpha, a hormone widely expressed in ovarian tissues and fallopian tubes, being responsible for stimulating uterine myometrium growth, preparing it for calving [34].

Identified for the second and the third calving, the windows region on BTA4 (34.9–35.6 Mb) (Table 1) encloses the gene *SEMA3D* (Semaphorin 3 D), which was differentially expressed in nervous system cells during the embryonic development of broilers [35]. The *SEMA3D* gene, in humans, is associated with Kallmann Syndrome, a condition characterized by delayed or absent puberty and an impaired sense of smell [36]. Also, this gene was associated with embryonic development in chickens [35] and with maternal calving difficulty in bovines [37].

The region BTA7: 106.8–107.0 Mb was associated to third, fourth, fifth and sixth calving. In this region is the *FBXL17* (F-Box and Leucine Rich Repeat protein 17) gene that is related to recycling processes in part of proteosoma 26 S. This gene was associated to conformation (morphological trait) in Nellore cattle [38] and transcript on skeletal muscle at different ages [39]. The BTA8:78.3–79.4 Mb region, associated to STAY in the fourth and fifth calving (Table 1), harbors the gene *TUT7* (terminal uridylyl transferase 7) that is involved with pos-transcriptional regulation of immune response cells (macrophages). After parturition, the cow is more susceptible to infections and diseases on genital tract such as ovarian and uterus [40] and the loss of immune response increases the animal susceptibility to diseases [41]. The *TUT7* gene also plays a role in oocyte maturation and fertility and is involved in microRNA induced gene silencing through uridylation of deadenylated miRNA targets [42].

Identified for the fifth and sixth calving, the BTA27: 2.12–21.8 Mb region overlapped QTLs associated to dystocia and calving ease [30]. This region encloses the *TUSC3* (tumor suppressor candidate 3) gene that was upregulated in placenta of women with HELLP syndrome which is associated with abortion and fetal morbidity [43]. The BTA19:43,9–44,3 Mb region was associated to STAY at the seventh and eighth calving (Table 1). The genes in this region are related to reproduction, energy metabolism and bone formation factors, which could be directly associated to cow’s longevity. The genes *ETV4* (ETS variant 4) and *ATXN7L3* (Ataxin 7 like 3), were differentially expressed in cattle oocytes during the folliculogenesis and ovulation, suggesting that these genes have a direct influence in females’ reproductive ability, especially during the formation and production of oocytes [44, 45]. The gene *ETV4* is crucial for embryonic stem cells properties such as proliferation and expression of stem cell-related genes [46]. The *SOST* (sclerostin) gene was widely associated with bone formation and density bone in humans [47–49], through expression of sclerostin and have a negative regulator of bone formation [49]. The *UBTF* (upstream binding transcription factor), is strongly associated with neurogenerative disease in childhood [50–52].

The region BTA11:4.1– 4.6 Mb, identified for the sixth and seventh calving, harbors the *TSGA10* (testis specific 10) gene that was associated with migration, differentiation, and cellular division in the initial phase of embryos development [53]. Associated to sixth and seventh calving, the BTA9:43.3–43.6 Mb region includes the *ATG5* (Autophagy related 5) gene that has an autophagic function, mainly in pre-implantation ovaries. This autophagic function activity was founded in abundance in ovaries with heat stress conditions in pigs [54]. This gene was associated with the development of embryos after four- and eight-cell stages in mouse [55]. The *RTN4IP1* (Reticulon 4 Interacting Protein 1) gene, also located in this genomic region, was related to breast cancer being upregulated in affected individuals compared to healthy [56, 57]. The BTA1:66.3–66.6 Mb was associated to STAY in the fifth, sixth and eighth calvings (Table 1). This region surrounds a QTL associated with puberty age and daily weight gain in bovine [30].

Seven of the identified genomic regions were specifically associated with STAY at the second calving (Table 1). Among these regions, the BTA2:135,4–136,1 Mb harbors the genes *MFAP2* (Microfibril associated protein 2) and *ATP13A2* (ATPase cation transporting 13A2). The *MFAP2* was previously related to infertility or failures in embryo implantation in humans [58]. The *ATP13A2* acts in the transport of zinc in nervous cells [59]. To remain in the herd, a cow must maintain a regular reproductive performance which can be directly influenced by the hormonal regulation acting on the pituitary-hypothalamus axis [60]. The BTA10:58–59 Mb region was associated with tick resistance in F1 Gyr X Holstein animals [61]. This region contains the genes *LYSMD2* (LysM domain containing 2) and *MYO5C* (Myosin VC). The *LYSMD2* was related to growth, cell adhesion and nervous signaling in live organisms [62]. A copy number variation in this gene sequence was associated, in humans, with aromatase excess syndrome, a disorder that causes prepubertal onset gynecomastia, hypogonadotropic hypogonadism, and short height in men. In women are usually asymptomatic, although macromastia, irregular menses, have been reported in a few individuals [63]. The *MYO5C* gene is related to the transport of melanin into melanocytes [64], and the degree of pigmentation is an important indicator of animal adaptation in tropical environments [65]. It is expected that females more adapted to tropical environments have longer reproductive life in the herd.

The BTA2:119.0–119.4 Mb (Table 1) was identified for the third calving, surround the genes *B3GNT7* (UDP-GlcNAc:BetaGal Beta-1,3-N-Acetylglucosaminyltransferase 7) and *NMUR1* (neuromedin U receptor 1). The *B3GNT7* was found co-expressed with genes related to a healthy human placenta with no possibility of premature calving [66]. The *NMUR1* was associated with environmental acclimation in sheep [67]. Also identified for the third calving, the BTA6:83.6–84.2 Mb region encloses the *TMPRSS11F* (transmembrane serine protease 11 F) gene, that was associated to immune response to mastitis [68] and somatic cell score [69] in dairy cattle and the BTA20:63.9–64.2 Mb region harbors the *SEMA5A* (semaphoring 5 A) gene, which was associated to antibody response in chickens [70]. All these results indicate the importance of adaptive traits for STAY mainly at the beginning of female’s reproductive life.

The region BTA8:45.9–46.3 Mb identified for the fourth calving, surround the *MAMDC2* (MAM Domain Containing 2) gene and was up regulated in endometrium from fertile women when were stimulated by progesterone [71]. This situation is common in beef cattle by time-fixed artificial insemination, widely used in the herds in our datasets. The *EYA2* (EYA Transcriptional Coactivador and Phosphatase 2) gene, located on BTA13:75.2–75,7 Mb, was reported as transcript on endometrium [72] and epithelial ovarian [73]. These findings suggest that reproduction factors have a major importance to keep a cow in the herd until the fourth calving.

A total of 35 regions were found to be associated to STAY in only one of the intermediate calvings (fifth or sixth). Identified for the fifth calving, the BTA9: 58.4–58.9 Mb harbors the 7SK gene that was associated to reproductive traits in horses [74], and beef cattle [75]. Moreover, the BTA12:60,7–61,4 Mb encloonses the HTATSF1 (HIV-1 Tat Specific Factor 1) gene that was related to estrogen receptor of precocious puberty women [76] and transcript in preimplantation of mouse embryos in 2-cell stage [77]. The *7SK* gene plays important role in growth control of primordial germ cells [78]. For the sixth calving, it was identified the BTA20:25.3–25.7 Mb region with the genes *FST* (Follistatin) and *NDUFS4* (NADH:Ubiquinone Oxidoreductase Subunit S4). The *FST* plays a role on formation of granulosa cells [79], having reduced expression in ovarian of pigs selected to increase ovulation rate. This gene was associated with heifer and cow conception rate [80]. The *NDUFS4* gene has been associated with progressive neurodegenerative disorder (Leigh syndrome) [81–85], which could lead to carriers’ death in initial period of life in humans and mouse. For the fifth and sixth calvings, our results showed genes with reproductive and nervous systems functions.

For STAY in the last calvings (seventh and eighth), 84 genes were identified. The BTA9: 41.3–41.8 Mb, associated with the seventh calving, harbors the *FOXO* (Forkhead Box) gene which was associated with longevity in humans [86] and in sheep [87]. This gene is related to apoptosis on granulosa cells and it was downregulated in primary ovarian when different levels of LH (Luteinizing hormone) and FSH (Follicle-stimulating hormone) concentration were used in chickens [88]. Associated to STAY in the eighth calving, the region BTA6:13.0–13.4 Mb, harbors genes such as: *TIFA* (TRAF interacting protein with forkhead associated domain), *ALPK1* (Alpha Kinase 1) and *FAM241A* (family with sequence similarity 241 member A). The *TIFA* gene was related with oxidative stress affecting the innate immune response [89]. Inflammatory mediators play a vital role in maintaining tissue homeostasis in the female reproductive tract, mainly during the ovulation process, which involves the rupture of the dominant follicle, and innate immunity and inflammation contribute to this process in the ovary [90]. The *ALPK1* gene was associated to adnexal carcinomas in reproductive system (ovaries and fallopian tubes) and tumor of cutaneous origin [91]. The *FAM241A* gene transcript in endometrium was related to reproductive disease and endometriosis in humans [92]. These last two genes show a function in reproductive performance. The BTA6: 15,0–15,5 Mb was also associated to STAY in the eighth calving. Located in this region, the ELOVL6 (ELOVL Fatty Acid Elongase 6) gene was associated to lipid’s metabolism in cattle [93], pigs [94, 95] and chickens [96]. Indeed, there is a close connection among fat metabolism, reproduction and lifespan, where alterations of fat content and/or composition are interconnected with reproductive system regulation contributing to extend longevity by influencing the overall metabolism of animals [97].

The list of genes identified in the present study was enriched for pathways related to feeding behavior (GO:0007631), digestion (GO:0007586), neuropeptide hormone activity (GO:0005184), G-protein coupled receptor binding (GO:0001664), regulation of appetite (GO:0032098) and Pancreatic hormone-like (IPR001955) (Table 2). Some of the genes involved in these pathways (PPY, PYY and PYY2) are related to energy metabolism, feed intake and satiety process through the metabolism of ghrelin hormone [98, 99]. The ghrelin hormone participates in the reproductive tissue hormonal regulation with positive feedback on pituitary and hypothalamus [100]. A modulation of PYY on the secretion of LH hormone was identified in mice [101]. These pathways affect animal’s reproductive performance by inducing the pituitary-hypothalamus axis to process reproductive hormone production. In humans, genes involved in these pathways also were related to increased food intake and elevated in nervous anorexy [102]. The related GO terms and pathways also refer to the importance of nutrition and digestion to STAY. Research has shown that inadequate nutrition can negatively impact the reproductive performance of dairy cows, including delayed onset of puberty, reduced conception rates, decreased pregnancy rates, and increased embryonic loss [103, 104]. As a quantitative and reproductive trait, STAY has strong influence from environment, with low heritability estimates [28]. Thus, our findings support the importance of nutrition and feed behavior on STAY at different calvings.

**Table 2:** Gene Ontology for genes associated to stayability considering different calvings in Nellore cattle

Term	Gene	*p*-value	Calving
IPR003877:SPla/RYanodine receptor SPRY	*TRIM41, TRIM7, HERC1, DDX1*	0.01700	2, 3, 7
IPR013320:Concanavalin A-like lectin/glucanase, subgroup	*TRIM41, EGFLAM, TRIM7, HERC1, DDX1, MAMDC2*	0.01700	2, 3, 4, 7
SM00449:SPRY	*TRIM41, TRIM7, HERC1, DDX1*	0.02000	2, 3, 7
IPR001870:B30.2/SPRY domain	*TRIM41, TRIM7, HERC1, DDX1*	0.02100	2, 3, 7
IPR001955:Pancreatic hormone-like	*PYY, PYY2, PPY*	0.00000	7, 8
GO:0032098 regulation of appetite	*PYY, PYY2, PPY*	0.00130	7, 8
GO:0005184 neuropeptide hormone activity	*PYY, PYY2, PPY*	0.01370	7, 8
GO:0007631 feeding behavior	*PYY, PYY2, PPY*	0.01650	7, 8
GO:0007586 digestion	*PYY, PYY2, PPY*	0.01920	7, 8
GO:0001664 G-protein coupled receptor binding	*PYY, PYY2, PPY*	0.02630	7, 8
GO:0051537 2 iron, 2 sulfur cluster binding	*ISCA1, XDH*	0.01490	4, 5, 7
Iron-sulfur	*ISCA1, XDH*	0.04683	4, 5, 7
IPR015943:WD40/YVTN repeat-like-containing domain	*SEMA5A, NBAS, HERC1, SEMA3D, RACK1, GNB5, STXBP5L*	0.03890	2, 3, 5, 6, 7, 8
transit peptide:Mitochondrion	*MRPL30, LIPT1, ISCA1, COX8B, NDUFS4, RTN4IP1*	0.03490	4, 5, 6, 7
Transit peptide	*MRPL30, LIPT1, ISCA1, COX8B, NDUFS4, RTN4IP1*	0.04799	4,5, 6, 7
IPR003954:RNA recognition motif domain, eukaryote	*NCL, PABPC1L, HTATSF1*	0.01490	3, 4, 5
SM00361:RRM_1	*NCL, PABPC1L, HTATSF1*	0.01710	3, 4, 5

Some of the enriched pathways (IPR003877: SPla/RYanodine receptor SPRY, IPR013320:Concanavalin A-like lectin/glucanase, SM00449:SPRY, IPR001870:B30.2/SPRY domain) shared TRIM family genes (Table 2) that have important function on elimination of shorted-lived regulatory proteins such as cell cycle regulation, cellular signalling and DNA repair and are related to development and progression of tumor [105]. These pathways also include the HERC family, which has been related to human diseases like cancer and neurological disorders [106]. Probably, these genes related to nervous system or neurological system could be associated to regulation of hormonal metabolites.

The concanavalin A-like domain, in bovine, plays an important role in sugar recognition and binding, and can have implications for milk production and immune defense [107]. This domain is a carbohydrate-binding found in a variety of proteins, including lectins, which are proteins that can bind to specific sugars. In bovines, the concanavalin A-like domain is found in several proteins, including the mannose-binding lectin (MBL), a serum protein that is part of the innate immune system and can recognize and bind to sugar molecules on the surface of pathogens, leading to their opsonization and clearance [107, 108].

The *SPRY* domain is a protein interaction module that is found in a wide range of eukaryotic proteins, with diverse functions [109]. It plays a critical role in several important signaling pathways, including RNA processing, regulation of histone H3 methylation, innate immunity, and embryonic development [110, 111].

Significantly enriched terms, families and pathways are GO:0051537 ~ 2 iron; 2 sulfur cluster binding; transit peptide:Mitochondrion; IPR015943:WD40/YVTN repeat-like-containing domain; IPR003954:RNA recognition motif domain; eukaryote; and SM00361:RRM_1. Iron is an essential molecule that plays a crucial role in cellular processes, including energy metabolism, DNA synthesis, and oxygen transport. The mitochondria play an essential role in iron metabolism, as they are involved in heme and iron-sulfur cluster biogenesis. In reproductive tissues, iron is necessary for gamete maturation and embryo development. During pregnancy, iron requirements increase due to the growth and development of the fetus, placenta, and expansion of the maternal blood volume [112, 113]. The RRM_1 domain is a highly conserved RNA-binding motif found in a variety of eukaryotic proteins involved in RNA metabolism [114]. Some studies suggest that RRM_1 may play a role in iron homeostasis through its regulation of iron-binding proteins. The transit peptide and RRM_1 domain contributes to the overall health and reproductive success of bovine, by facilitating proper mitochondrial, RNA function, and iron metabolism [115]. Any disruption in these processes can lead to negative effects on reproductive functions.

## Conclusions

Many of the genomic regions identified in this study were associated to STAY in more than one calving, indicating that part of the genetic control of this trait acts throughout the female’s productive life. The *in silico* functional analyses of the genes found in this study, indicate that while the expression of stayability at earlier ages (from the 2th to the 6th calving) appeared to be more influenced by genetic factors linked to reproductive performance together with an overall health/immunity, at later ages (7th and 8th calvings) genetic factors related to an overall animal health gain more relevance. These results could support that selecting for stayability at earlier ages (perhaps at the second calving) should be applied instead of selecting for this trait at later ages, having practical implications in breeding programs since it could drastically reduce the generation interval, accelerating the genetic progress.

## Data Availability

The data that support the findings of this study are available from Alliance Nellore breeding program (https://gensys.com.br) but restrictions apply to the availability of these data, which were used under license for the current study, and so are not publicly available. Data are however available from the authors (L.G. Albuquerque, galvao.albuquerque@unesp.br) upon reasonable request and with permission of Alliance Nellore breeding program (https://gensys.com.br).
