# Correction: Genome-wide association study for stayability at diferent calvings in Nellore beef cattle

**DOI:** 10.1186/s12864-024-10098-4

**Published:** 2024-02-26

**Authors:** Diogo Osmar Silva, Gerardo Alves Fernandes Júnior, Larissa Fernanda Simielli Fonseca, Lúcio Flávio Macedo Mota, Tiago Bresolin, Roberto Carvalheiro, Lucia Galvão de Albuquerque

**Affiliations:** 1 Animal Science Department, School of Agricultural and Veterinary Sciences, São Paulo State University (Unesp), Jaboticabal, SP Brazil; 2 National Council for Scientifc and Technological Development (CNPq), Brasília, Brazil; 3 Departamento de Zootecnia, Via de acesso Paulo Donato Castellane s/n, CEP: 14884-900 São Paulo, Jaboticabal, Brazil

**Correction:** ***BMC Genomics*** **25, 93 (2024)**

10.1186/s12864-024-10020-y

Following publication of the original article it was reported that the names of Gerardo Alves Fernandes Júnior and Lucia Galvão de Albuquerque were incorrectly published as ‘Gerardo Alves Fernandes’ and ‘Lucia Galvão Albuquerque’ respectively.

The original article has been updated.

